# Curvature-driven spatial patterns in growing 3D domains: A mechanochemical model for phyllotaxis

**DOI:** 10.1371/journal.pone.0201746

**Published:** 2018-08-16

**Authors:** Mara D. Rueda-Contreras, José R. Romero-Arias, José L. Aragón, Rafael A. Barrio

**Affiliations:** 1 Instituto de Neurobiología, Universidad Nacional Autónoma de México, Juriquilla, Querétaro 76230, Mexico; 2 Centro de Física Aplicada y Tecnología Avanzada, Universidad Nacional Autónoma de México, Juriquilla, Querétaro 76230, Mexico; 3 CONACYT - Instituto de Física y Matemáticas, Universidad Michoacana, Ciudad Universitaria, Morelia, Michoacán 58040, Mexico; 4 Instituto de Matemáticas, Universidad Nacional Autónoma de México, Campus Juriquilla, Juriquilla, Querétaro 76230, Mexico; 5 Instituto de Física, Universidad Nacional Autónoma de México, 01000 Ciudad de México, Mexico; University of California San Diego, UNITED STATES

## Abstract

Here we discuss the formation of phyllotactic patterns in the shoot apical meristem (SAM) of plants, where the spatial distribution of the phytohormone auxin determines phyllotaxis in a domain that is growing and changing in time. We assume that the concentration of auxin modifies the mechanical properties of the domain and that the mechanical stress field in the SAM orients the flux of auxin. To study this problem we propose a mechanism for pattern formation in growing domains with variable curvature. The dynamics of chemicals is modeled by a reaction-diffusion system that produces a three dimensional pattern of chemical concentrations that changes the stress field in the domain while growing. The growth process is modeled by a phase-field order parameter which determines the location of the boundaries of the domain. This field is coupled to the chemical concentration through a curvature term that affects the local mechanical stress in the domain. The local stress changes in turn modify the chemical patterns. Our model constitutes a useful and novel approach in theoretical biology, as many developmental processes in organisms seem to be affected by the changes of curvature, size, mechanical stress and other physical aspects. Several patterns seen in many plants are reproduced under certain conditions by our model.

## Introduction

Phyllotaxis is the arrangement of repeated plant organs such as leaves, floral structures, ribs in cacti or scales in a pine. This geometrical patterning has fascinated minds for centuries. The scientific importance of studying the form of living organisms was pertinently pointed out by Darcy Thompson [1]. Phyllotactic patterns can be obtained with very simple space filling physical rules, as in foams [2] and Bénard convection cells [3].

The development of form, or *morphogenesis* was a term coined by Alan Turing in his seminal article [4], where he pointed out that the diffusion of reacting chemicals should be essential to produce stationary stable spatial patterns. At the end of his life Turing worked with the problem of Fibonacci Phyllotaxis from the reaction-diffusion point of view and left an unpublished manuscript entitled “Outline of the development of the daisy”, with key ideas and suggestions [5].

Pattern formation on evolving domains has been widely studied. Reaction-diffusion pattern formation on one-dimensional growing domains was first studied in [6–8]. The study was then extended to growing planar domains [9] and continuously growing surfaces [10–12]. A further generalization to evolving curved surfaces was natural due to the application to growing biological organisms [13–20].

Beyond these studies, a topic of great current interest is the role of mechanical forces during pattern formation. In his pioneering work Turing recognized that a complete model of a growing embryo should include the mechanical and the chemical parts but, for the sake of mathematical simplicity, the mechanical aspects were ignored and the chemical part is the most significant in his theory [4].

Chemical theories about phyllotaxis appeared due to Alan Turing’s reaction-diffusion model of morphogenesis, which prompted a number of works based on diffusing morphogens capable of produce phyllotactic patterns [21, 22].

Mechanical and mechanochemical approaches in biological pattern formation began in the eighties (see for instance Ref. [23, Ch.6] and references therein) but it is still a problem of great interest in several biological realms. Embryology, phyllotaxis, tumor formation, angiogenesis and cell motility are examples where the influences of mechanical forces can not be dismissed. Models integrating mechanical and chemical aspects to explain pattern formation are still rare (for a review see Ref. [24]). It is worth mentioning a model for embryonic pattern formation [25], where the tissue curvature and morphogen expression are coupled in a positive feedback loop; the domain is considered as a thin film and the model is based on an energy minimization approach.

In recent years there has been an enormous advance, experimental [26, 27] and theoretical [28–30], in the biology of the early development of meristems in plants. The unveiling of the role played by the pytohormone auxin on plant development has opened an interdisciplinary way to approach this question [31–34]. Some authors have obtained phyllotactic-like patterns on the basis of a more realistic set of hypothesis that incorporate main biological facts, such as polar auxin transport in a growing structure [35, 36]. These, however, did not consider mechanical or geometrical influences due to plant growth itself, which strongly affect this process [37–40]. Many fundamental questions of plant development that have remained unanswered, such as positioning of lateral organs around the root [41], organization of plant tissues to resist environmental stresses [42] or the choice of the oriented cell division planes [43–45], seem to have a common basis: the response of plant cells to mechanical stress and the feedbacks involved in this response [46]. It has been found that the geometry and mechanics of the shoot apical meristem (SAM), the place where phyllotaxis occurs, affect the distribution of auxin. The hormone, on the other hand, has an influence on the mechanical properties of the SAM. The modification of these properties allows for growth, and this growth feeds back the way auxin is redistributed [46–48].

The incorporation of the biomechanics in models started more recently [39, 49, 50]. Furthermore, it has been also taken into account that the transport of auxin is facilitated by PIN1 proteins [32], to propose even more realistic models. From these, it is worth mentioning three discrete ODE models proposed in 2006 [35, 36, 51]. The first one was recasted using a continuous approximation, producing a partial differential equation model where the surface deformation is slaved to the auxin field [48]. It was shown that this model produces the spiral phyllotaxis properties observed on plants [52]. It is worth mentioning that the continuum approximation of a discrete model of polar auxin transport and cell dynamics [35], where the surface deformation is slaved to the auxin field, is the basis of a recent model that produces beautiful phyllotactic patterns on a planar disk geometry [52].

Here we retake the reaction-diffusion approach to propose a simple model, under the assumption that the important biological fact is that local auxin concentration differences are essential to regulate growth and that growth modifies the mechanical stresses that govern the redistribution of auxin as microtubules orient along stress fields [26] and there is a significant correlation between PIN localisation and microtubule orientation [34]). The proposed model couples chemical reaction-diffusion processes with the curvature and mechanical stress of a 3-dimensional domain in a reciprocal manner: the concentration of chemicals modifies the stress field of a tissue, and in turn, the chemical diffusion is driven by the stress field. This interplay occurs in a growing and evolving domain, whose evolution depends, likewise, on the distribution of the chemical. Thus the model includes an explicit interplay between the stress tensor, the curvature, and the distribution of a chemical modifying the mechanical properties of the domain. Also, these interactions are based on the complex dynamics of auxin in the SAM, which affect and are affected by the mechanics of growth [34, 53].

The chemical aspect of the model is accounted by a reaction-diffusion system coupled with the mechanical part by means of tensor diffusion coefficients that depend on the stress tensor. In this sense, chemicals are diffused directionally, leaded by the mechanics of growth.

The mechanical part of the model includes growth and it is carried out by a phase-field model, which is a mesoscopic version of the Ginzburg-Landau approach. Phase-field models, which constitute a mathematical method to study interfacial problems, have demonstrated to be successful tools in modeling dynamical processes in biology [54, 55]. We model growth and deformation as a phase-field whose evolution, and consequently its mechanical properties, depend on the concentration of the chemicals.

The model is therefore divided into two parts. The first one accounts for the deformation and growth of a closed surface, within which a chemical is diffusing. The growth of this surface is determined by the distribution of the chemical on it. These two processes are modeled by the so called phase-field equations. The evolution of the surface is described by a dynamical equation obtained from a free energy functional, whereas the distribution of the chemical is governed by a second dynamical equation obtained from the same energy density so, the two processes are necessarily coupled. The dynamical equation for the chemical is understood as a directional diffusion process, leaded by the mechanics of growth. We shall call this set of equations the *mechanical model*.

The second part of our model will account for the dynamics of the chemicals as a Turing process, in which two morphogens react and diffuse. We will refer to this second set of equations as the *chemical model*. Thus, these two parts constitute a mechano-chemical model for pattern formation.

## Methods

### The models

Phase-field models are mathematical devices for solving interfacial problems, in which the boundary conditions for the interface are substituted by a partial differential equation that tracks the evolution of an auxiliary field (the phase-field) *ϕ*. This field is a smooth function $\phi :\Omega \subset R^{3}\to R$ that acts as an order parameter that defines two stable phases. In the Ginzburg-Landau approach [56] one considers two domains, an inner fluid and an (aqueus) outer environment, and associates each domain to one of the equilibrium phases where *ϕ* takes the respective stable values, typically +1 and −1. The two domains are connected by a smooth interface of width *ϵ*, in which *ϕ* changes abruptly from one phase to the other. This interface is usually supposed to be located at the level set *ϕ* = 0. Thus, we model the surface of the SAM as a phase-field *ϕ* whose evolution depends on the concentration of a chemical *u*, which is also modelled as a conservative field. Furthermore, the evolution of *u* depends on the stress field generated by *ϕ* and it is composed by two parts: usual Fickean diffusion and active transport directly oriented by the stress. We achieve this modelling of active transport by defining a diffusion coefficient that depends on the stress tensor *σ*_*αβ*_.

The chemical substance *u* promotes the surface growth by enhancing the local curvature and thus changing the geometrical and mechanical properties of *ϕ*, *i.e.*, its stress and strain. For this reason, the equation for the energy density of the whole system includes a spontaneous curvature term that depends on *u*. This term allows that small surface deformations emerge, so one can interpret this effect as an increment on the surface elasticity. This is done because, in the context of phyllotaxis, auxin modifies the mechanical properties of the plant cell walls by enhancing cell wall elasticity, trigering the leaf formation [57–63]. Furthermore, the changes on the local curvature feed back the evolution of *u* by changing the stress field on *ϕ*. The mechanics of surface growth and the dynamics of the chemical substance are thus coupled in the equation of the free energy of the system. We now introduce the derivation of the phase-field model.

#### The mechanical model

Phase-field models have their origins in the Landau theory [56], where the energy of a system is expanded by powers of a scalar field called order parameter and usually denoted by *ϕ*. This expression defines the free energy of the system F, which is the internal energy of the system minus the amount of energy that cannot be used to perform any work. Ginzburg and Landau generalized this approach by incorporating powers of the order parameter gradient into the free energy:

$$\begin{array}{c} F=\int L(\phi ,\nabla \phi ,\nabla^{2}\phi )dV, \end{array}$$ (1)

where L is the energy density of the system. Because of the symmetry, homogeneity and isotropy of the system, the energy density can be written as $L=\Phi^{2}\left[\phi \right]$ where the functional is [64]:

$$\begin{array}{c} \Phi \left[\phi \right]=-\phi +\phi^{3}-\epsilon^{2}\nabla^{2}\phi . \end{array}$$ (2)

Note that the function *f*(*ϕ*) = −*ϕ* + *ϕ*^3^ is homogeneous and bistable, which is appropriate as *ϕ* must have two stable phases.

The chemical potential *μ*[*ϕ*] associated with the Cahn-Hilliard problem (the free energy change from deviations in the concentration of a species from a reference value) can be obtained as the functional derivative of the free energy [55]:

$$\begin{array}{c} \mu \left[\phi \right]=\frac{\delta F}{\delta \phi }≐\frac{\partial L}{\partial \phi }-\nabla_{\alpha }\frac{\partial L}{\partial (\nabla_{\alpha }\phi )}+\nabla^{2}\frac{\partial L}{\partial (\nabla^{2}\phi )}. \end{array}$$ (3)

Here we use spatial (Cartesian) coordinates so *α* = 1, 2, 3, and the subscripts on the gradient operator refer to differentiation with respect to the corresponding coordinate.

The minimum of the free energy (1) is obtained by setting Φ[*ϕ*] = 0, which can be solved by adding the boundary conditions at the bulk phase *ϕ*(**x**) → ±1 as **x** → ±∞. In this way one obtains the equilibrium profile of the interface, that turns out to be a smooth tanh-like function with an interface width *ϵ*. The chemical potential is important because, as we will show, it governs the time evolution of the order parameter.

We now add a spontaneous curvature term in order to incorporate the influence of the chemical *u*. This can be done by adding an extra term in the free energy density so the new free energy functional will be

$$\begin{array}{c} F_{SC}=\int_{\Omega }\Phi_{SC}^{2}\left[\phi \right]dV, \end{array}$$ (4)

where

$$\begin{array}{c} \Phi_{SC}\left[\phi \right]=\Phi \left[\phi \right]-\epsilon C_{0}(\phi^{2}-1). \end{array}$$ (5)

The term *C*_0_ is related to the spontaneous curvature of *ϕ*, and the term (1 − *ϕ*^2^) acts as a *δ* function centered at the interface, so it confines this effect to the surface *ϕ* = 0 and has no effect in the bulk phases. The interface is thus forced to accommodate its surface to the spontaneous curvature *C*_0_. It is important to mention that this phase-field model can be obtained from the formulations of the Canham-Helfrich problem [65] and both models are equivalent [54]. The Canham-Helfrich problem describes the evolution of interfaces governed by bending energy. This local bending energy is an expansion it terms of the two curvature invariants of a surface: the mean and the Gaussian curvatures. The elastic energy of the surface is expressed in this way, and it characterizes the relaxation of the surface towards stationary shapes. With no constraints and no preferred curvature, a surface would relax to a state with zero curvature, so it is possible to find stationary shapes of a surface under constraints, such as a constant volume enclosed by the surface or a fixed surface area [55]. Here, we impose the appearance of a spontaneous curvature on the surface.

The spontaneous curvature can be position-dependent or even *ϕ*-dependent. We assume that the surface changes its curvature in response to the concentration of the chemical substance, in a similar way as it is known to occur in phyllotaxis: the relaxation of the surface rigidity allows for the emergence of a small protuberance (a *primordium* in the phyllotaxis jargon) that will develop into a new organ, and it necessarily changes the curvature locally [66]. Thus, we set *C*_0_ as a quadratic functional of the chemical concentration *u*:

$$\begin{array}{c} C_{0}=C_{0}\left[u\right]=\beta u^{2}. \end{array}$$ (6)

Here *β* is the strength of the influence of chemical substance *u* on the tissue curvature. A quadratic dependence of the form (6) is the simplest assumption to asses this process because the interface grows as *R*^2^, where *R* is the radius of the apical dome. The free energy of the system with the coupling of the chemical substance then reads

$$\begin{array}{c} F_{SC}=\underset{\Omega }{\int }{\left(\left(\phi^{2}-1\right)\left(\phi -\epsilon \beta u^{2}\right)-\epsilon^{2}\nabla^{2}\phi \right)}^{2}dV. \end{array}$$ (7)

Finally, we include the terms of surface tension of *u* and *ϕ*, that is ∣ ∇*ϕ* ∣^2^ and ∣ ∇*u* ∣^2^, to the energy density so we can model the energetic cost of the surface relaxation. The complete free energy reads

$$\begin{array}{c} F=\int_{\Omega }(\Phi_{SC}^{2}\left[\phi \right]-\frac{1}{2}\varrho_{\phi }|\nabla \phi {|}^{2}-\frac{1}{2}\varrho_{u}|\nabla u{|}^{2})dV, \end{array}$$ (8)

where $\varrho_{\phi }$ and $\varrho_{u}$ are positive constants. Surface is created preferably in places where the concentration of *u* is large, thus it is reasonable to expect that the surface tension coefficient $\varrho_{\phi }$ should be much larger than $\varrho_{u}$. This is also inspired by phyllotaxis as it is known that the epidermal layer of the SAM (called L1) has large stiffness [26].

Once *ϕ* and *u* are coupled in the energy density, we should establish their time evolution. As we are assuming that the material is locally conserved through time, the dynamic evolution of *ϕ* is determined by Cahn-Hilliard dynamics [54, 64, 67]. Thus, one should first know how the free energy of the system changes respect to *ϕ* and this change is then subjected to diffusion. If *D*_*ϕ*_ and *D*_*u*_ are diffusion coefficients for *ϕ* and *u*, their dynamics is governed by (Ref. [54])

$$\begin{array}{ccc} \frac{\partial \phi }{\partial t} & = & \nabla \cdot (D_{\phi }\nabla \frac{\delta F}{\delta \phi }), \end{array}$$ (9)

$$\begin{array}{ccc} \frac{\partial u}{\partial t} & = & \nabla \cdot (D_{u}\nabla \frac{\delta F}{\delta u}). \end{array}$$ (10)

Now, since growth and deformation occur, and since cell proliferation and elongation take place during phyllotaxis, the amount of material is necessarily variable. We then need to introduce an additional term which adds mass to the domain. As the auxin promotes the tissue growth in plants, we add a term to the dynamic equation of *ϕ* which depends on the concentration *u*. Then, Eq (9) becomes

$$\begin{array}{c} \frac{\partial \phi }{\partial t}=D_{\phi }\nabla^{2}(\frac{\delta F}{\delta \phi })+\alpha \left[u\right], \end{array}$$ (11)

where *α*[*u*] = *mu*^2^ is a quadratic functional that adds mass to *ϕ*, and *m* is the amount of added mass per unit time. The introduction of *α* allows for a plausible modelling of growth and not only deformation of a volume under stress.

In most higher plants the SAM is an axisymmetric dome-like structure which exhibits specific growth patterns. In the peripheral zone, where the new primordia emerge, the cells have higher growth rates and are displaced radially into the flanks of the meristem, creating a basipetal flow of cells that induces a tip growth [66]. For modelling the characteristic tip growth of the SAM we use the premise that the region where primordia emerge has an increased mitotic activity, which is related to auxin concentration [66, 68]. We propose that this can be modelled by means of a normal distribution of auxin centered at the axis of the dome. Thus, *ϕ* is initialized as an axisymmetric dome and we introduce such a Gaussian distribution *G*[*u*], with a variable height that changes according to the displacement of the summit of the dome. This distribution is included in the dynamic Eqs (10 and 11) so they become

$$\begin{array}{ccc} \frac{\partial \phi }{\partial t} & = & D_{\phi }\nabla^{2}(\frac{\delta F}{\delta \phi })+\alpha \left[u\right]+\kappa mG\left[u\right], \end{array}$$ (12)

$$\begin{array}{ccc} \frac{\partial u}{\partial t} & = & \nabla \cdot (D_{u}\nabla \frac{\delta F}{\delta u})+G\left[u\right], \end{array}$$ (13)

where *κ* > 0.

Now, as the main hypothesis of this work is that the dynamics of *u* on *ϕ* is directed by the stress field, we establish an explicit dependence of the diffusion coefficient of *u* on the stress tensor *σ*_*αβ*_. Thus, we define a tensorial diffusion coefficient

$$\begin{array}{c} D_{u}=\gamma \sigma_{\alpha \beta }, \end{array}$$ (14)

where *γ* > 0. In this way Eq (10) becomes

$$\begin{array}{c} \frac{\partial u}{\partial t}=\gamma \nabla \cdot (\sigma_{\alpha \beta }\nabla \frac{\delta F}{\delta u})+G\left[u\right]. \end{array}$$ (15)

A brief description on how to compute the stress tensor within this framework is given in Ref. [55]. In terms of the variations of the free energy (1) one has

$$\begin{array}{c} \sigma_{\alpha \beta }=(L-\phi \frac{\delta L}{\delta \phi })\delta_{\alpha \beta }-\frac{\partial L}{\partial (\nabla_{\beta }\phi )}\nabla_{\alpha }\phi +\nabla_{\beta }\frac{\partial L}{\partial (\nabla^{2}\phi )}\nabla_{\alpha }\phi -\frac{\partial L}{\partial (\nabla^{2}\phi )}\nabla_{\alpha }\nabla_{\beta }\phi . \end{array}$$ (16)

An explicit calculation of Eq (16), valid for any free energy of the form (1), is presented in S1 Appendix of the Supporting Information. Thereby, the stress tensor in the whole volume depends explicitly on the distribution of *u* and in the absence of a chemical substance, it is computed directly by the energy density of *ϕ* alone. Thus, both *u* and *σ*_*αβ*_ undoubtedly play a principal role in the phase field dynamics and the evolution of SAM shape.

The first term of Eq (16) is related to the hydrostatic pressure, as it is diagonal, and the second and third terms are associated with the surface tension of *ϕ*. Furthermore, the curvature tensor *Q*_*αβ*_ is contained in the third term of Eq (16) (see S1 Appendix of the Supporting Information). Finally, the geometry of the interface deformation is implicitly contained in the gradients of *ϕ*. Thus, the mechanical properties of the volume and the interface are completely determined from the free energy and its dependence on spontaneous curvature.

By performing the functional derivative in Eqs (10) and (11) we obtain the dynamic equations for *ϕ* and *u*, which determine the tissue growth and the auxin distribution on it. Variations of the energy with respect to *ϕ* and *u* are

$$\begin{array}{ccc} \frac{\delta F}{\delta \phi } & = & 2[(3\phi^{2}-1-2\phi \epsilon \beta u^{2})\Phi_{SC}\left[\phi \right]-\epsilon^{2}\nabla^{2}\Phi_{SC}\left[\phi \right]]+\varrho_{\phi }\nabla^{2}\phi , \end{array}$$ (17)

$$\begin{array}{ccc} \frac{\delta F}{\delta u} & = & -4\epsilon (\phi^{2}-1)\beta u\Phi_{SC}\left[\phi \right]+\varrho_{u}\nabla^{2}u. \end{array}$$ (18)

Then, inserting these expressions in (11) and (15), we obtain the master equations for modeling the coupled processes of growth, deformation and the distribution of the chemical *u* on the surface *ϕ*:

$$\begin{array}{ccc} \frac{\partial \phi }{\partial t} & = & D_{\phi }\nabla^{2}\{[2(3\phi^{2}-1-2\phi \epsilon \beta u^{2})\Phi_{SC}\left[\phi \right]-\epsilon^{2}\nabla^{2}\Phi_{SC}\left[\phi \right]]+\varrho_{\phi }\nabla^{2}\phi \}+\\ & & \alpha [u]+\kappa mG[u], \end{array}$$ (19)

$$\begin{array}{ccc} \frac{\partial u}{\partial t} & = & \gamma \nabla \cdot (\sigma_{\alpha \beta }\nabla \{-4\epsilon (\phi^{2}-1)\beta u\Phi_{SC}\left[\phi \right]+\varrho_{u}\nabla^{2}u\})+G\left[u\right]. \end{array}$$ (20)

These equations are obtained from the simplest assumptions that can be made in the framework of the phase-field theory for this complex process. The minimization of the free energy determines how the surface moves and how the chemical substance will be distributed in space. Also, these two processes are coupled, and both are linked in a feedback loop by the energy density in Eq (8).

Eqs (19) and (20) model the mechanical part of the process of surface growth and deformation, which should be coupled with the chemical part, described below.

#### The chemical model

In this work it is assumed that a chemical substance *u* is involved in the phase field dynamics through a curvature term (see Eq 6). We assume that this substance interacts with a second substance (morphogen) *v* forming a reaction-diffusion system. In phyllotaxis, *u* is clearly identified with auxin. The other morphogen *v* might be any yet unknown substance whose only requirement is that it interacts with the morphogen *u* [35, 36, 69]. By not compromising our more fundamentally mechanical model to the action of a particularly biological substance, the morphogen *v* might play the role of cytokinin, a hormone that is implicated in antagonistic interactions with auxins [70]. Auxin rapidly down regulates biosynthesis of cytokinin, which is a candidate for the factors that control auxin receptivity in the SAM [70].

Our main assumption here is that the reaction-diffusion process is faster than the growth and deformation of the surface. Thus, the reaction-diffusion system, which is capable to produce stationary spatial patterns (through a diffusion driven or Turing instability) will provide almost stationary distributions of the chemical while the surface is changing (see the next Section for details).

Turing equations describe the time evolution of the concentration of two or more substances, called morphogens, that diffuse and react according to nonlinear kinetics. A substance *u* is thus allowed to interact with another morphogen *v* and the two morphogens diffuse with different rates. For the non-linear kinetics, we adopt the so-called BVAM model [71, 72], which has proved to be useful to model many different biological processes. The choice of the BVAM model was basically made because it is considered a generic model assuming mass conservation, not tailored to a particular biological mechanism. This model has also proved to present a wealthy variety of behaviors [71–76].

The BVAM kinetics is obtained by assuming that there is a fixed point at (*U*_*c*_, *V*_*c*_) and Taylor-expanding around this fixed point, keeping terms up to third order. One defines *u* = *U* − *U*_*c*_ and *v* = *V* − *V*_*c*_, so there is a uniform stationary solution at the point (*u*, *v*) = (0, 0). In non dimensional form the model reads,

$$\begin{array}{ccc} \frac{\partial u}{\partial t} & = & D\nabla^{2}u+\eta (u+av-cuv-uv^{2}), \end{array}$$ (21)

$$\begin{array}{ccc} \frac{\partial v}{\partial t} & = & \nabla^{2}v+\eta (bv+hu+cuv+uv^{2}). \end{array}$$ (22)

Here, *D* is the ratio of the diffusion coefficients *D*_*u*_/*D*_*v*_. The special arrangement of the coefficients *a*, *b*, *c* and *h* follows from conservation relations between chemicals, and *η* is a scaling parameter.

In the next Section we explain how the dynamical Eqs (19 and 20) and the Turing system (21 and 22) are related. Basically, the Turing mechanism is introduced as *a pulse* while the dynamical equations are solved.

Since we are interested in applying our model for generating phyllotactic patterns, we take advantage on the analyses of the BVAM model that can predict different symmetries when solved on disks [73] or spheres [77]. However, it is important to note that this model is used to feed the mechanical model with symmetric patterns in three dimensions, as it does not generate phyllotaxis de novo. The relations between symmetric solutions and the Turing instability analysis are worked out in S1 Appendix of the Supporting Information.

### Computational details

The chemical model, Eqs (21) and (22), provides the initial pattern of auxin *u*, which is the input of the mechanical model, Eqs (19) and (20). As the mechanical model evolves, however, the chemical process is still working in the evolving domain *ϕ*, as detailed below.

All the partial differential equations that must be solved in our model are highly non-linear, so a numerical method is needed to integrate them. For the spatial dependence we used standard second-order finite differences, and for the time dependence we used a simple Euler method. Their convergence was first tested by generating patterns with the symmetry predicted by the spherical harmonics analysis (see S1 Appendix of the Supporting Information).

Considering that the chemical reactions are very fast processes, while growth can take days or even months to occur, we solve the equations as follows: as the Turing model, Eqs (21) and (22) attains a stable stationary state very rapidly, we integrate it for a few iterations with time scale *dt*_1_ = 5 × 10^−2^, and then solve the mechanical model, Eqs (19) and (20) for a long time, with time scale *dt*_2_ = 1 × 10^−5^. This allows the mechanical model to accommodate to the conditions yielded by the chemical model. These time scales ensure that the dynamics of change in shape is 5000 times slower than the dynamics of the morphogens *u* and *v*, defined in this three dimensional space. Also, we used grid parameters *Nx* = *Ny* = 40, *Nz* = 60, and a space step *dx* = 1. We imposed zero-flux boundary conditions on the surface *ϕ* = 0.

The parameters of the chemical model were chosen as in [72, 73, 77] in order to obtain the Turing instability: *a* = 1.1123, *b* = −1.0122, *h* = −1 and *D* = 0.516 for all the simulations. The parameter *c* was varied in the range 0 ≤ *c* ≤ 0.57 to obtain different types of patterns. The parameter *η*, which represents the size of the domain for a Turing system, was varied first, in order to obtain the distinct spherical harmonics on the sphere (see S1 Appendix of the Supporting Information). Actually, the symmetries of the Turing patterns on the sphere and the disk depend only on the domain size [73, 77]. These symmetric patterns are however stationary, so they cannot be considered phyllotactic patterns. When the chemical and mechanical models are coupled, *η* was chosen according to the spherical harmonic that it produces on the sphere (see Fig A in S1 Appendix of the Supporting Information). Most of the experimental results in which this particular simulations are based come from the flowering plant *Arabidopsis thaliana*. As all the tissues of a young *Arabidopsis* plant are capable to contribute to synthesize auxin [68], we started our simulations with a random distribution of *u* and *v*, which is a perturbation around the steady state (0, 0) of the chemical model.

**Fig 1:**
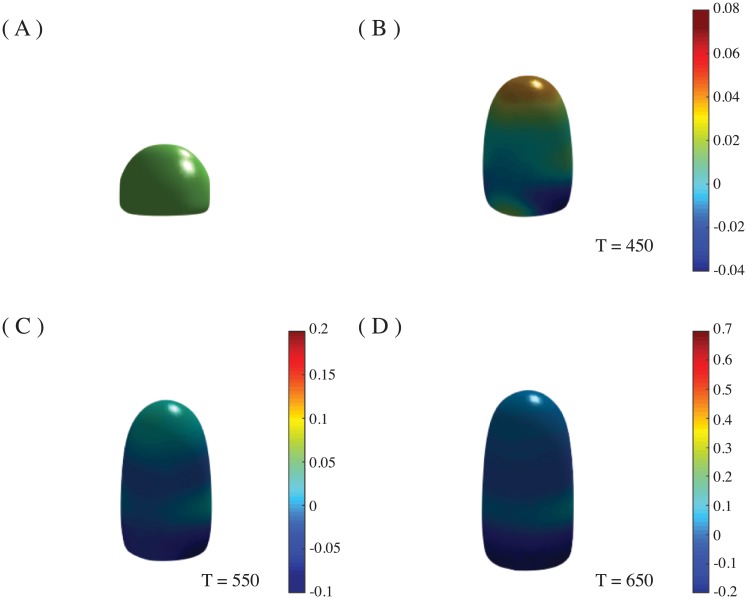
Initial domain and growth pattern. (A) Initial domain: a cylinder of radius *R* = 10 and height *H*_*z*_ = 6, with a hemispherical tip. (B)-(D) Growth patterns of the domain (tip growth). The Gaussian distribution *G*[*u*] has a width of *R*/2 and it can be seen at the tip in (B) and (C). Images are taken at times T = 450, 550, 650.

For the application to phyllotactic pattern formation, we defined an initial surface *ϕ*_0_ that consists of a cylinder of radius *R* = 10, with a dome-like tip (the SAM), approximated by a hemisphere (Fig 1A). This surface is where *ϕ* = 0 and divides the inner region (where the phase field is *ϕ* = 1) and the outer region (*ϕ* = −1), and it was allowed to evolve according to Eqs (19) and (20). The cylindrical part of the dome has height *H*_*z*_, which was fixed to *H*_*z*_ = 6 but, as we included an explicit tip growth for the dome in the simulations, different heights can be reached as the equations are solved (Fig 1B–1D). The surface tension coefficients were fixed to $\varrho_{\phi }=2.5$, $\varrho_{u}=0.5$. Finally, *D*_*ϕ*_ = 1 and *m* = 15 were set for all the simulations. The different phyllotactic patterns obtained depend on the values of *η*, *γ*, and *β*. That is, the parameter that determines the spherical harmonics for the Turing system, the influence of the stress strength on the auxin flux, and the strength of the auxin influence on the local curvature.

The Gaussian distribution *G*[*u*] was centered at $\left(\frac{Nx}{2},\frac{Ny}{2},H_{z}\right)$, with *H*_*z*_ varying with growth, so the distance of the source to the uppermost point of *ϕ*_0_ remains constant. The width of this Gaussian is related to *R*, and it turns out to be important for the pattern formation. The characteristic tip growth of the SAM is obtained by means of this normal distribution (Fig 1B–1D). The parameter *κ*, which allows for non-constant addition of mass into *G*[*u*] (Eqs 12 and 13), is varied in order to obtain different patterns (*κ* = 1, 2). It is important to mention that without this explicit tip growth no phyllotactic patterns were obtained but only symmetrical ones.

As the young primordia exhibit the highest capacity to synthesize auxin *de novo* and contain almost a ten times more auxin concentration than any other tissue [68], we fixed the emerging primordia as relative sources of auxin. This is achieved by adding constant terms into the dynamic Eqs (19 and 20), which are different from zero only in the place where a primordium is present. This was done to maintain the position of the primordia along the simulations once they are formed.

## Results

**Fig 2:**
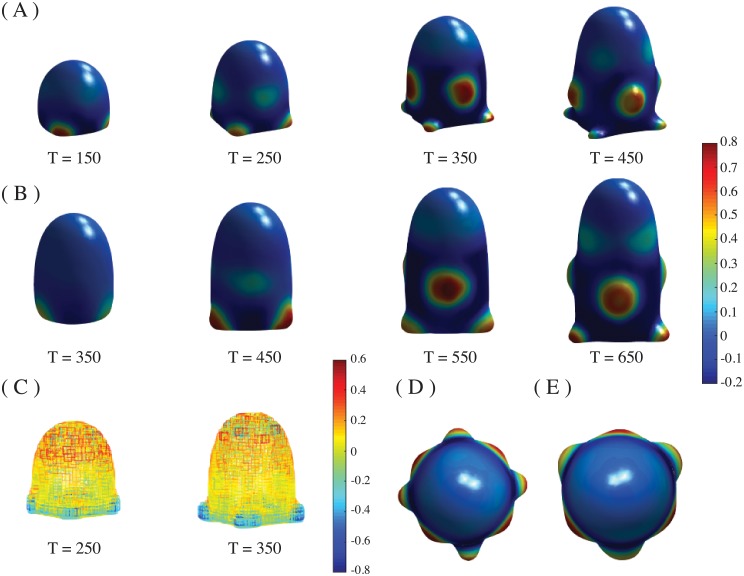
Whorled phyllotactic patterns. Time sequence of emergence of primordia into whorled phyllotactic patterns. Parameter values are *β* = 0.5, *γ* = 0.2, *c* = 0.57, *G*[*u*] with a width of *R*/2, *κ* = 2, and the rest of parameters as indicated in the text. The whorls appear in the regions where the mean curvature changes more abruptly. (A) Four-whorled phyllotactic pattern, *η* = 0.3902. Images are taken at times T = 150, 250, 350, 450. (B) Three-whorled phyllotactic pattern, *η* = 0.2601. Images are taken at times T = 350, 450, 550, 650. (C) Mean curvature of the domain at times T = 150 and T = 350 for the 4-whorled pattern evolution shown in (A). (D)-(E) Top view of (A) and (B) at times T = 350, and T = 550, respectively.

With all these assumptions and sets of parameters we obtained phyllotactic-like patterns on the growing dome. With the guidance of the spherical harmonics produced by the Turing system (*c* = 0.57, *c* = 0.33) we were able to obtain 2, 3, 4, 5 and 6-whorled phyllotaxis, up to four plastochrones (four generations of a whorl). Whorled phyllotactic patterns with symmetries 4 and 3 are shown in Fig 2A and 2B and are obtained with the values *γ* = 0.2, *β* = 0.5, and a Gaussian distribution *G*[*u*] of width *R*/2. The first whorl emerges at the basis of the dome, which is the region where the curvature of the domain changes more abruptly (Fig 2C).

This is in agreement with experimental observations which indicate that the primordia emerge exactly where the curvature of the SAM dome changes [78]. As the dome grows due to the Gaussian distribution of auxin fixed at the tip, primordia continue to emerge in the available region with more pronounced changes in curvature. It is noticeable from Fig 2C that the next generation of primordia will emerge where there is a major change of curvature from zero to a positive value. This behaviour continues for all the generations of primordia. From the fourth plastochrone a deformation begins near the base and the phyllotactic pattern becomes distorted. Thus, whorled phyllotactic patterns are formed in the correct spatial and temporary order, up to four generations of a whorl.

Whorled patterns with symmetries 2, 5 and 6, as well as the videos of the complete simulations up to T = 1000 time iterations, including the curvature time evolution, are included as Supporting Information. All the whorled patterns are obtained basically with the same parameters of Fig 2A and 2B, except for the value of the parameter *η* (see Fig A and Equations C and D, in S1 Appendix of the Supporting Information).

**Fig 3:**
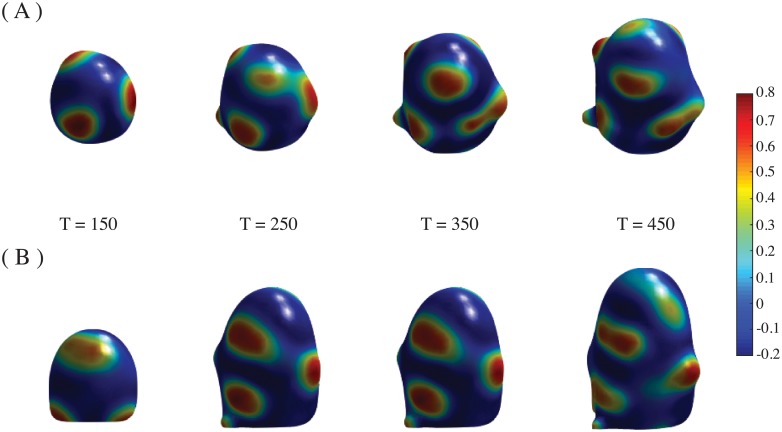
Aberrant patterns. Patterns formed with no stress or spontaneous curvature. (A) Time evolution of the pattern shown in Fig 2A, but with values *β* = 0, *γ* = 0. (B) Same as (A), but only *β* = 0 (*γ* = 0.2 as in Fig 2A). Images are taken at times T = 150, 250, 350, 450.

The perpetuation of the symmetrical whorl to form the phyllotactic pattern is due to the phase-field mechanism. In particular, it is a result of the stress-dependence of the auxin transport as well as the influence of auxin on the spontaneous curvature (see Fig 3 and S9 Video of the Supporting Information).

**Fig 4:**
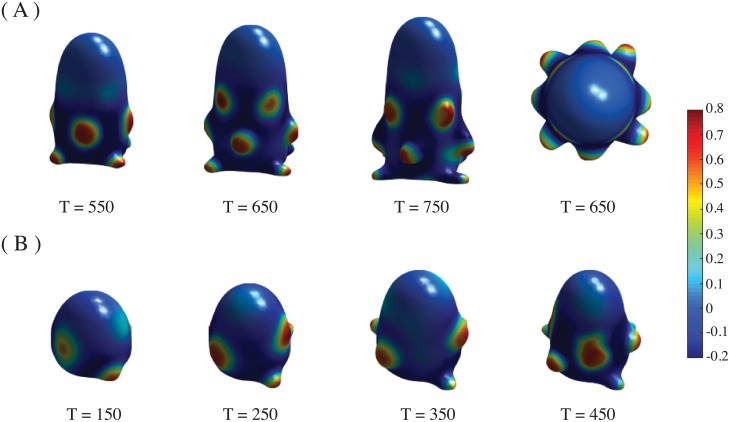
Aberrant patterns. Patterns formed with high stress and a wider Gaussian distribution *G*[*u*]. (A) Time evolution of the pattern shown in Fig 2A, but with *γ* = 3.5. Compare the top right panel, taken at time T = 650, with Fig 2B. (B) Same as Fig 2A, but the width of *G*[*u*] is *R*/2.5.

The patterns are not correctly formed either when *γ* or *β* are zero (Fig 3). Coefficients *γ* and *β* can vary within certain ranges to produce phyllotactic patterns. Outside these ranges the patterns become aberrant (Fig 4A).

**Fig 5:**
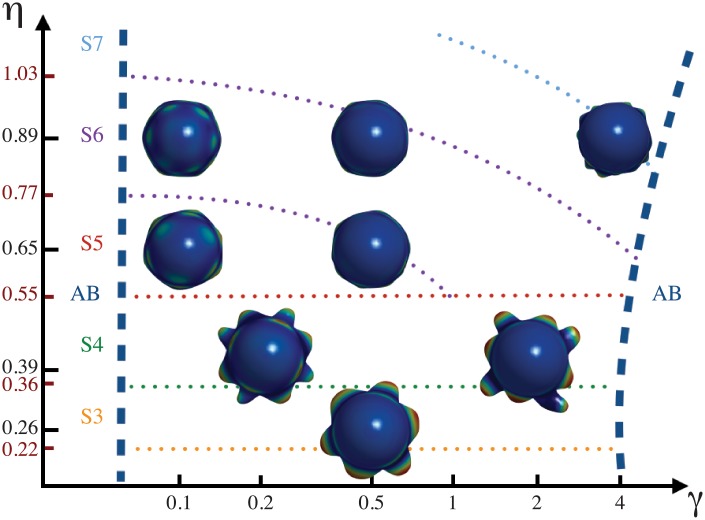
Phase diagram for whorled patterning. Whorled phyllotactic patterns emerge as a function of the stress tensor coupling. When *γ* increases the emergence of primordia is delayed and the whorls become distorted. The symmetry of whorled patterns increases as as function of *η*. The dotted lines represent the boundary limits for 3- to 7-symmetry (S3, S4, S5, S6, S7) and the numerical limit values are indicated in red on *η* axis. Note that in some cases, the increment of *γ* maintains the symmetry (S3, S4, S5, S6) but it augments the symmetry in other cases (S5 → S6, S6 → S7). The dash blue lines delimitate the whorled and aberrant patterns (AB). The values of *η* for each image are indicated on axis (in black) and the rest of the parameter values are *β* = 0.5, *G*[*u*] with a width of *R*/2, *κ* = 2 and *c* = 0.33 for *η* = 0.65 and *η* = 0.89; and *c* = 0.57 for *η* = 0.26 and *η* = 0.39.

**Fig 6:**
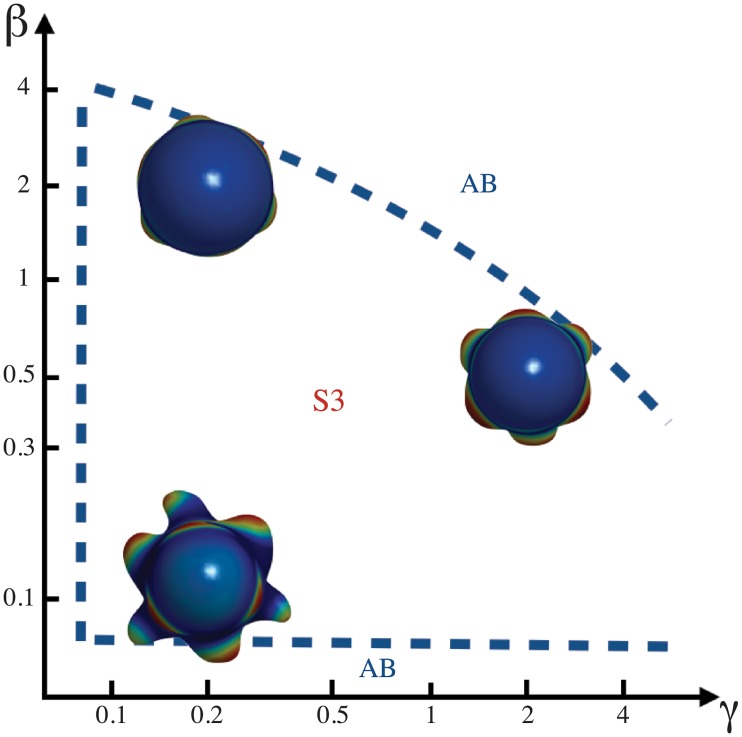
Phase diagram for 3-symmetry whorled patterning. Whorled phyllotactic patterns change with spontaneous curvature interaction *β* and stress tensor coupling *γ*. When *γ* increases *β* decreases in order to maintain the emergence of primordia as whorled patterns. Aberrant patterns (AB) emerge outside of the dash blue lines. Parameter values are *η* = 0.26, *G*[*u*] with a width of *R*/2, *κ* = 2 and *c* = 0.57.

A phase diagram for whorled phyllotactic patterns is shown in Fig 5. Whorled phyllotaxis emerges as a function of the stress coupling *γ*. As *γ* increases, the emergence of primordia is delayed and thus the patterns become aberrant. The stress coupling has also an influence on patterns with higher symmetry, that is, 5 and 6. Furthermore, the influence of *u* on the elasticity of the domain, *β*, decreases as the stress coupling increases, thus maintaining the whorled patterns. This behavior is illustrated in Fig 6 for 3-whorled phyllotaxis and it is the same for all the other symmetries. Outside the regions indicated in these diagrams the patterns become aberrant. This suggests that alterations of the mechanical stress in the SAM could be used to modify phyllotactic patterns in a predictable way. The width of the distribution *G*[*u*] turns out to be also important for the formation of phyllotactic patterns (Fig 4B), thus suggesting that the mitotic activity of the SAM must be a highly regulated process.

The need of fine tuning of *G*[*u*] to obtain regular geometric patterns could be justified in terms of the biological characteristics of the meristematic system. The SAM is divided into two functional zones according to its growth patterns. The central zone (CZ), located at the center of the dome near the tip, contains the stem cells which provide new cells to keep the plant growing. Daughter cells are displaced into the peripheral zone (PZ) at the flanks of the dome, where the new primordia emerge [66]. Thus, the PZ has a different mitotic activity than the rest of the structure, as it is composed of fast growing cells which induce a basipetal flow of cells from the top of the dome [66].

This flow is necessarily constrained by the dimensions of the SAM and any other physical or biological influences, so the tip growth must be defined in agreement with these influences. We chose the width of the Gaussian to be *R*/2 because all the information of a normal distribution is within two standard deviations. This value turned out to be the suitable one to form the phyllotactic patterns. It is important to say that some authors suggest that a meridional gradient of extensibility is the key mechanical feature that explains the tip-growth morphogenesis among many taxonomic groups [79]. The introduction of this Gaussian distribution of extensibility is also a key feature in our model.

**Fig 7:**
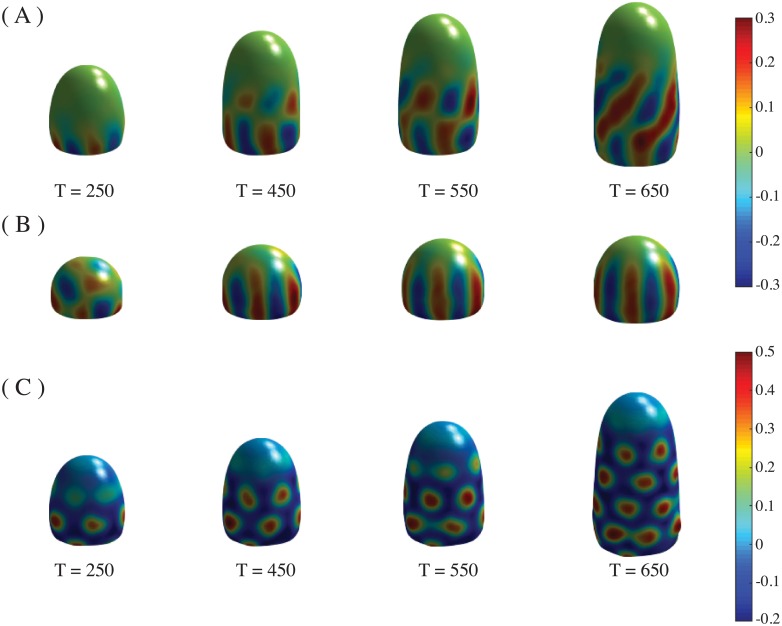
Spiral patterns. Time sequence of emergence of spiraling patterns. Parameter values are *β* = 0.5, *γ* = 0.2 and the rest of parameters as indicated in the text. (A) Spiral pattern with 5-fold symmetry, *η* = 0.5676, *κ* = 2, *c* = 0 and *G*[*u*] with a width of *R*/2. Images are taken at indicated times. (B) Ribs pattern with 6-fold symmetry, *η* = 0.6504, *κ* = 0.5, *c* = 0 and *G*[*u*] with a width of *R*/1.5,. Images are taken at indicated times. (C) Five-whorled phyllotactic pattern, *η* = 0.8437, *κ* = 2, *c* = 0.33 and *G*[*u*] with a width of *R*/2. Packing of primordia into parastichies can be seen at time iteration T = 500 and on.

When *c* = 0 a different phyllotactic pattern is obtained, with symmetrical ribs along the dome, as in cacti (Fig 7A and 7(b)). It is noteworthy that this *c* value produces stripes instead of spots in a Turing system [72]. With *c* = 0.33, the 5, and 6-whorled phyllotactic patterns are obtained, but as the simulation time reaches *T* = 500 the whorls become to be packed into spirals or, in the phyllotaxis jargon, parastichies (Fig 7C). If we adopt the naming of phyllotactic patterns based on the number of parastichies, a (3, 4)-phyllotaxis is obtained. This value of *c* is just within the range of the Turing region [72]. This type of phyllotactic pattern is perpetuated up to five plastochrones. The correct temporal and spatial emergence of these patterns also depends on the values of *γ* and *β*.

**Fig 8:**
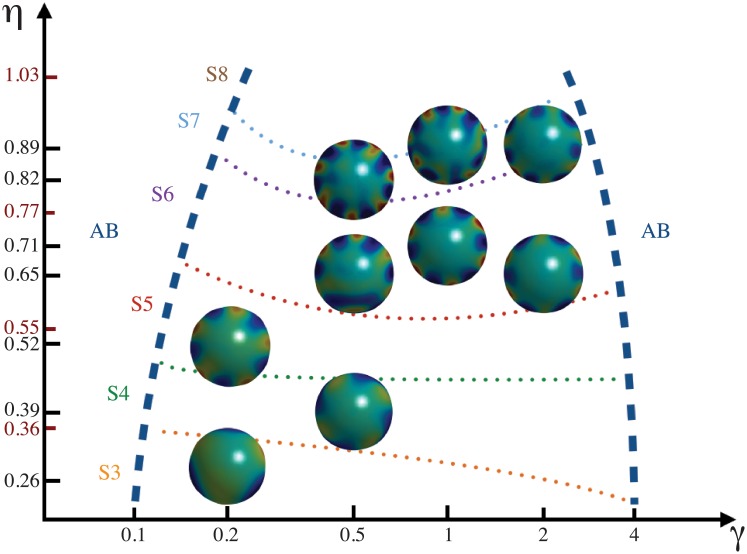
Phase diagram for ribbed patterning. Ribbed patterns emerge as a function of the stress tensor coupling. The symmetry of ribs increases as a function of *η*. The dotted lines represent the boundary limits for 3- to 8-symmetry (S3, S4, S5, S6, S7, S8) and the numerical limit values are indicated in red on *η* axis. Note that in all cases the symmetry of the pattern is below of the limit value, which means that in some cases, the stress coupling *γ* raises the symmetry. Emergence of aberrant patterns (AB) is indicated with dashed blue lines. The values of *η* for each image are indicated on *η* axis (in black) and the rest of the parameter values are *β* = 0.5, *G*[*u*] with a width of *R*/1.5, *κ* = 0.5 and *c* = 0.

A phase diagram is shown in Fig 8. As in the case of whorled phyllotaxis, ribbed patterns emerge as a function of the stress coupling, which also influences the symmetry of the pattern. Outside the ranges shown in Fig 8 the patterns become aberrant. It is noteworthy that the lower the stress the higher the symmetry. This fact explains the cacti patterning, which has a nearly spherical shape: higher symmetries are allowed in their ribs as the stress is distributed homogeneously on a sphere. We thus see that the stress and curvature-driven transport of the morphogen *u*, as well as the tip growth of the SAM dome, are sufficient to reproduce a variety of stable phyllotactic patterns.

**Fig 9:**
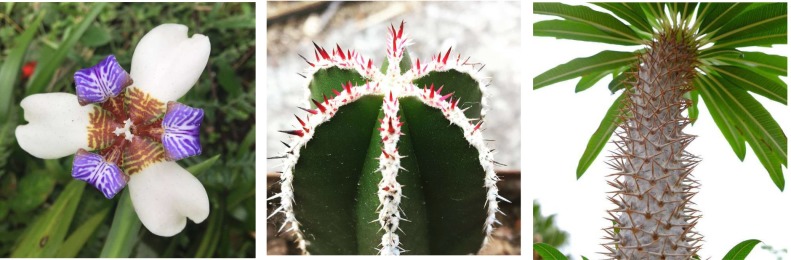
Phyllotaxis. (a) Three-whorled phyllotactic pattern as in Fig 2(e); the different floral organs emerge in time as whorls of three. (b) Phyllotactic pattern of ribs with 6-symmetry as in Fig 7(b). (c) Phyllotaxis of *Pachypodium lamerei*; plants of this genus exhibit the pattern shown in Fig 7(c): their leaves first emerge as whorls and then pack into parastichies.

In Fig 9 we show examples of different phyllotactic pattern found in plants in order to compare them with some of the numerical calculations for our model.

## Discussion

Based on the phase-field approach we constructed a mathematical model for pattern formation on domains that change in size and shape in the course of time. The domain evolves according to the minimization of a free energy that is constructed on the basis of the bending energy of a surface, but also as a result of the influence that a chemical substance has on its mechanical properties. This is inspired in the process of phyllotactic pattern formation in plants, where the fitohormone auxin is the implicated chemical substance. Our model reproduces phyllotaxis and it is supported by the experimental finds of the roles that auxin, in conjunction with the mechanics of the shoot apical meristem (SAM), play in this developmental process.

The main assumptions of our model are based on the biology of phyllotaxis and they are basically two: the first one is that a chemical substance enhances the elasticity of the evolving surface and the second is that the distribution of the chemical substance on the surface is in turn guided by the stresses that the surface exerts while growing. The first assumption is accomplished by introducing a spontaneous curvature term in the free energy of the system, which depends on the concentration of the chemical. Thus, the more chemical is present on the surface, the easier it is deformed. This is based on the fact that the auxin promotes growth in plants by reducing the stiffness of the cell walls [57–59]. Thus, a new plant organ primordium can emerge where auxin accumulates because the surface of the SAM yields to pressure and allows for a rapid deformation. The second assumption is achieved by making the diffusion of the chemical to be dependent on the stress tensor, which determines the magnitudes and directions of the stresses on the surface. This dependence was chosen to be linear for simplicity, and it is based on the way that auxin is distributed in the SAM surface while phyllotaxis occurs.

Auxin flow is a combination of diffusion, as well as an active transport mediated by the membrane proteins PINFORMED1 (PIN1) [32]. It has been verified that these proteins orient themselves towards the sites of emergence of new primordia in L1 [32], so this active transport gives directionality to the flux. The mechanism of PIN1 polarization is still unknown but it has been observed that it is oriented parallel to the alignment of cortical microtubules in the SAM [34]. Microtubules in turn tend to align in the direction of maximal stress operating in cell walls [26, 34, 66]. We summarize all these experimental observations by stating that the stresses on the surface orient auxin flux. Our tensor equation does this job and these two particular assumptions are sufficient to generate phyllotaxis. The rest of the assumptions and proposed interactions are purely physical, so the model can be used to simulate the development of many other living systems and this patterning mechanism could be considered as fundamental (see S9 Video of the Supporting Information).

The novelty of these findings is that the emergence of phyllotactic patterns in three dimensions is completely due to mechanical interactions. The regions where the auxin accumulations sites emerge is a consequence of the phase-field mechanism. These regions are the ones in which the curvature of the domain changes more abruptly. This is reasonable, as the curvature tensor *Q*_*αβ*_ is present on the stress tensor, so the tip growth provides new available space with changing curvature, and the tensor diffusion is *choosing* the place where primordia should appear. Moreover, the stress-driven flux of the chemical is responsible for the perpetuation of the patterns. Furthermore, primordia do not emerge in other regions on the domain, in particular, they do not appear in the dome summit, which in plants is a region where the auxin has no effects. Our model thus suggests that the emergence of the distinct functional regions of the SAM could emerge as natural consequences of the mechanics of growth, which can be used to regulate the molecular and genetic activity in plants as we have shown in [69].

**Fig 10:**
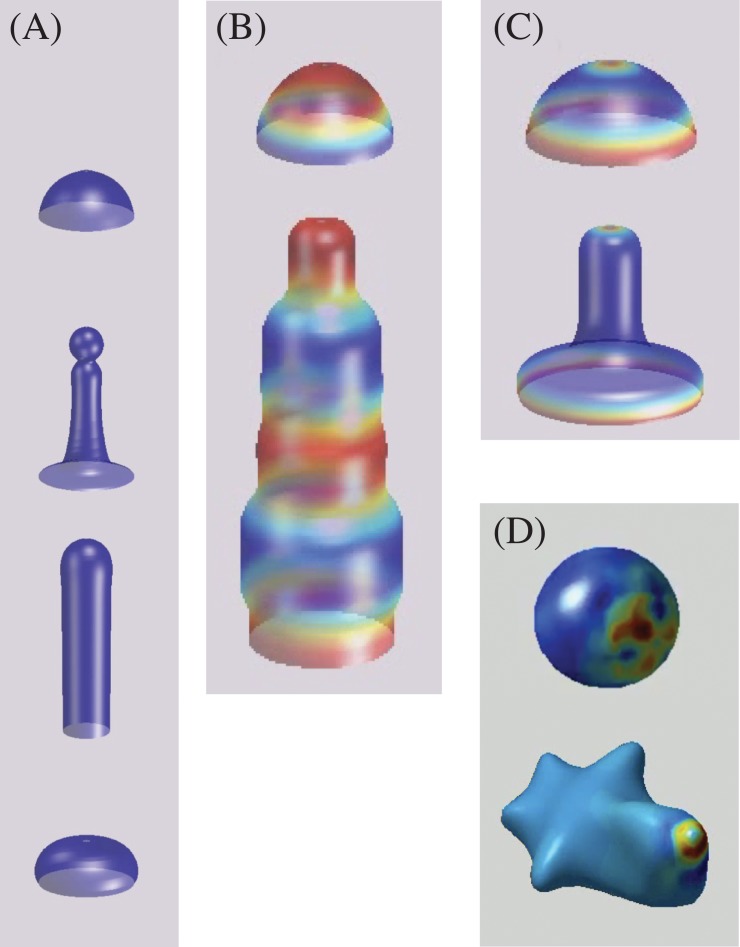
Mechanical model without the interaction with the chemical model. Shapes obtained starting with an initial hemisphere defining *ϕ* = 0, and using various stationary forms for the field *u*. (A) Field *u* is a Gaussian centered at the symmetry axis with increasing width. From top to bottom, width is 0, *R*/4, *R*/2 and *R*. (B) Field *u* on the surface follows a fixed sinusoidal vertical pattern. (C) A two dimensional *u* fixed field with maxima at the tip and at the bottom of the initial domain. (D) Gaussian distribution of *u* with center off the symmetry axis. There is no Turing mechanism in (A), (B) and (C).

We have circumscribed the presentation of our model to phyllotaxis in plants, and using knowledge of parameters suitable to *Arabidopsis* to obtain some specific results. However, the basic physical mechanisms in our model could be acting in other problems of morphogenesis as well. In order to illustrate this statement in Fig 10 we show the growing shapes obtained with our mechanical model using different conditions for the *u* field, and maintaining the parameters for *ϕ* constant. It is remarkable the variety of shapes that one could obtain with this very simple model.

We can conclude that any developmental process in living systems should be supported and sustained on physics. There appear new interactions and interactors in each developmental stage of any living system, so it is adequate to model such processes as blocks or modules sustained on physical interactions that must be well known.

## Supporting information

S1 Appendix Curvature tensor, dynamical equations and stability of Turing patterns. (PDF)

S1 Video Emergence of primordia with 3-fold symmetry. Corresponding to Fig 2B and 2E of the main text.
(MP4)

S2 Video Emergence of primordia with 4-fold symmetry. Corresponding to Fig 2A and 2D of the main text.
(MP4)

S3 Video Emergence of ribs with 6-fold symmetry. Corresponding to Fig 7A of the main text.
(MP4)

S4 Video Emergence of ribs with 6-fold symmetry. Corresponding to Fig 7B of the main text.
(MP4)

S5 Video Emergence of primordia with 5-fold symmetry. Corresponding to Fig 7C of the main text.
(MP4)

S6 Video Emergence of primordia into whorled phyllotactic pattern with 2-fold symmetry. Parameter values are *β* = 0.5, *γ* = 0.2, *G*[*u*] (width of *R*/2), *κ* = 2, *c* = 0.57, *η* = 0.2014. The remaining parameters are indicated in the main text.
(MP4)

S7 Video Emergence of primordia into whorled phyllotactic pattern with 5-fold symmetry. Parameter values are *β* = 0.5, *γ* = 0.2, *G*[*u*] (width of *R*/2), *κ* = 2, *c* = 0.33, *η* = 0.6504 and the rest of parameters as indicated in the main text.
(MP4)

S8 Video Emergence of primordia into whorled phyllotactic pattern with 6-fold symmetry. Parameter values are *β* = 0.5, *γ* = 0.2, *G*[*u*] (width of *R*/2), *κ* = 2, *c* = 0.33, *η* = 0.8919 and the rest of parameters as indicated in the main text.
(MP4)

S9 Video Principal directions of stress tensor in whorled phyllotactic patterning with 3-fold symmetry. In the top we plotted front and top views of the principal directions of the stress tensor (black arrows) in the volume. In the bottom, the principal directions of stress (black arrows) are plotted only in the surface. The domain was plotted in diffused color to identify the regions that are growing. The color scale indicates *u* concentration.
(MP4)
