# Supplementary material for: Curvature-driven spatial patterns in growing 3D domains: A mechanochemical model for phyllotaxis
Source: PLoS One. 2018 Aug 16;13(8):e0201746. doi: 10.1371/journal.pone.0201746 (PMC6095518; doi:10.1371/journal.pone.0201746)
Supplement: S1 Appendix — (PDF) [file pone.0201746.s001.pdf]

Supporting Information for *Curvature-driven spatial patterns in growing 3D domains: a mechanochemical model for phyllotaxis*

Mara D. Rueda-Contreras[1,4], José R. Romero-Arias[2,3], José L. Aragón[4,*], Rafael A. Barrio[5]

1 Instituto de Neurobiología, Universidad Nacional Autónoma de México, Campus Juriquilla, Boulevard Juriquilla 3001, Juriquilla, Querétaro 76230, Mexico
2 CONACYT - Instituto de Física y Matemáticas, Universidad Michoacana, Edif. C-3, Ciudad Universitaria, Morelia, Michoacán 58040, Mexico
3 Instituto de Matemáticas, Universidad Nacional Autónoma de México, Campus Juriquilla, Boulevard Juriquilla 3001, Juriquilla, Querétaro 76230, Mexico
4 Centro de Física Aplicada y Tecnología Avanzada, Universidad Nacional Autónoma de México, Campus Juriquilla, Boulevard Juriquilla 3001, Juriquilla, Querétaro 76230, Mexico
5 Instituto de Física, Universidad Nacional Autónoma de México, Apartado Postal 20-364, 01000 Ciudad de México, Mexico

*** jlaragon@unam.mx**

S1 Appendix

Curvature tensor and dynamical equations

The total free energy of the system is:

$$\mathcal{F} = \int_{\Omega} \left(\Phi_{SC}^2[\phi] - \frac{1}{2}\varrho_{\phi}|\nabla\phi|^2 - \frac{1}{2}\varrho_{u}|\nabla u|^2 \right) dV,$$

where ϱ_{ϕ} and ϱ_{u} are the surface tension constants and the chemical potential Φ_{SC} is:

$$\Phi_{SC}[\phi] = \left(\phi^2 - 1\right)\left(\phi - \epsilon\beta u^2\right) - \epsilon^2\nabla^2\phi.$$

Here u represents the auxin concentration (which, as it has been shown, also plays the role of a spontaneous curvature). The variation of the free energy respect to ϕ can be computed to the first three terms as:

$$\frac{\delta\mathcal{F}}{\delta\phi} = \frac{\partial\mathcal{L}}{\partial\phi} - \nabla_{\alpha}\cdot\frac{\partial\mathcal{L}}{\partial(\nabla_{\beta}\phi)} + \nabla_{\alpha\alpha}\frac{\partial\mathcal{L}}{\partial(\nabla_{\beta\beta}\phi)}.$$

Spatial Cartesian coordinates $\alpha = 1, 2, 3$ are used, so the subscripts α, β in ∇ refer to differentiation with respect to the corresponding coordinate.

If we consider that $\phi(\mathbf{x}) \equiv \tanh(d(\mathbf{x})/\epsilon)$, where $d(\mathbf{x})$ is the distance to the interface [7], then:

$$\nabla_{\alpha}\phi = \frac{(1-\phi^2)}{\epsilon}\hat{e}_{\alpha},$$

where direction vector is obtained by $\hat{e}_{\alpha} = \nabla_{\alpha}d(\mathbf{x})$. Based on this calculation it is easy to show that the parametrization of ϕ gives the the unit normal vector to the interface as the first derivative, that is:

$$\nabla d(\mathbf{x}) = \hat{n}.$$

With these ideas in mind and after some algebra we can prove that [3, 7]:

$$\nabla_\alpha \nabla_\beta d(\mathbf{x}) = \frac{\epsilon}{1-\phi^2}\left[\nabla_\alpha \nabla_\beta \phi + \frac{2\phi}{1-\phi^2}\nabla_\alpha \phi \nabla_\beta \phi\right] \equiv Q_{\alpha\beta},$$

where $Q_{\alpha\beta}$ is the three dimensional curvature tensor.

This curvature tensor has five remarkable properties, which couples the volume with the surface. These properties are: (1) the tensor is symmetric: $Q_{\alpha\beta} = Q_{\beta\alpha}$, (2) the curvature tensor has a zero eigenvalue and therefore its determinant is zero, (3) the invariants of the tensor can be expressed by the coefficients of the characteristic polynomial, (4) the coefficients correspond to the determinant, the trace, and the sum of the principal minors of the tensor matrix, and (5) the non-vanishing invariants are related to the mean $\mathcal{H}$ and Gaussian $\mathcal{K}$ curvatures of the surface [8] as:

$$\begin{aligned}
\mathcal{H} &= \frac{1}{2}\mathrm{Tr}[Q_{\alpha\beta}], \\
\mathcal{K} &= \sum_{\alpha\beta}\left[\left(Q_{\alpha\alpha}Q_{\beta\beta} - Q_{\alpha\beta}^2\right)\frac{1-\delta_{\alpha\beta}}{2}\right],
\end{aligned}$$

respectively, and $\delta_{\alpha\beta}$ is the Kronecker delta.

On the other hand, the stress tensor defined in [2] by:

$$\sigma_{\alpha\beta} = \left(\mathcal{L} - \phi\frac{\delta\mathcal{F}}{\delta\phi}\right)\delta_{\alpha\beta} - \frac{\partial\mathcal{L}}{\partial(\nabla_\beta\phi)}\nabla_\alpha\phi + \nabla_\beta\frac{\partial\mathcal{L}}{\partial(\nabla_{\alpha\alpha}\phi)}\nabla_\alpha\phi - \frac{\partial\mathcal{L}}{\partial(\nabla_{\alpha\alpha}\phi)}\nabla_\beta\nabla_\alpha\phi,$$

can be written after some algebra as:

$$\sigma_{\alpha\beta} = P_0\delta_{\alpha\beta} + g_6\hat{e}_\alpha\hat{e}_\beta - g_7\epsilon\beta u\nabla_\beta u\hat{e}_\alpha - g_8 Q_{\alpha\beta} + g_9\epsilon\beta u^2 Q_{\alpha\beta},$$

where

$$P_0 = g_0 + g_1\epsilon\beta u^2 - g_2\epsilon^2\beta^2 u^4 + g_3\epsilon\beta u\nabla u\cdot\hat{n} - g_4\epsilon\beta|\nabla u|^2 - g_5\epsilon\beta u\nabla^2 u,$$

is the hydrostatic pressure, $\hat{n}$ is a unitary normal vector and the functions g_j depend only on ϕ and ϵ, as:

$$\begin{aligned}
g_0 &= (\phi^2-1)\left(\phi^2(1-5\phi^2) - 2\phi\epsilon^2\nabla^2\phi\right) + 2\phi\left(5\phi^2-1\right)\epsilon^2\nabla^2\phi - \frac{1}{2}\varrho_\phi\left(|\nabla\phi|^2 + 2\phi\nabla^2\phi\right) - \\
&\quad 2\phi\epsilon^4\nabla^4\phi + \epsilon^4(\nabla^2\phi)^2, \\
g_1 &= (\phi^2-1)\left(8\phi^3 - 4(\phi^2-1) - 2(2\phi^2-1)\epsilon^2\nabla^2\phi\right) - 4\phi^2\epsilon^2\nabla^2\phi, \\
g_2 &= (\phi^2-1)(3\phi^2+1), \\
g_3 &= (\phi^2-1)(16\phi^2\epsilon), \\
g_4 &= (\phi^2-1)(4\phi\epsilon^2) + \frac{1}{2}\frac{\varrho_u}{\epsilon\beta}, \\
g_5 &= (\phi^2-1)(4\phi\epsilon^2), \\
g_6 &= (\phi^2-1)\left(\frac{\varrho_\phi}{\epsilon^2}(\phi^2-1) + 2(\phi^2-1)^2 + 2\phi\epsilon^2\nabla^2\phi\right), \\
g_7 &= (\phi^2-1)\left(4\epsilon(\phi^2-1)\right), \\
g_8 &= (\phi^2-1)\left(10\phi\epsilon(\phi^2-1) - 2\epsilon^3\nabla^2\phi\right), \\
g_9 &= (\phi^2-1)\left(2\epsilon(\phi^2-1)\right).
\end{aligned}$$

It is noteworthy that all the functions g_j have the factor (ϕ^2-1), which means that the stress tensor acts mainly in the vicinity around the interface, as it can be expected.

Stability of Turing patterns

For the chemical model we use a general phenomenological Turing system based on the BVAM model [4]. The general non-dimensional form of this system describes the spatial and temporal variations of the concentrations u and v as:

$$\frac{\partial u}{\partial t} = D\nabla^2 u + \eta\left(u + av - cuv - uv^2\right), \tag{A}$$

$$\frac{\partial v}{\partial t} = \nabla^2 v + \eta\left(bv + hu + cuv + uv^2\right), \tag{B}$$

where D is the ratio of the diffusion constants for u and v and η is related to the domain size. We consider unitary domains of size $L = 1$ and scale this dimension as $\eta = L^2/a\delta$ for some scale factor δ. The constants a, b, c and h are the parameters of the Turing kinetics. The appropriate parameter values resulting in the Turing instability and the stability of the stationary states result from the calculation of the characteristic polynomial:

$$|\mathbf{A} - \mathbf{D}k^2 - \lambda\mathbf{I}| = 0,$$

where k is the wave mode and:

$$\mathbf{A} = \eta\begin{pmatrix} 1 - v^2 - cv & -2uv + a - cu \\ v^2 + h + cv & b + 2uv + cu \end{pmatrix}_{(u_0, v_0)},$$

$$\mathbf{D} = \begin{pmatrix} D & 0 \\ 0 & 1 \end{pmatrix}.$$

The stationary solution (u_0, v_0) with $h = -1$ is around $(0, 0)$ and the dispersion relation for the BVAM model is:

$$\lambda^2 + \left[(1 + D)k^2 - \eta(1 + b)\right]\lambda + Dk^4 - \eta k^2(Db + 1) + \eta^2(b - ah) = 0.$$

The unstable wave modes can be estimated by means of the critical value of the bifurcation parameter by noticing that at the onset of the instability $\lambda(k_c) = 0$, so:

$$Dk_c^4 - k_c^2(Db + 1) + \eta^2(b - ah) = 0.$$

This result gives the most unstable wave number when:

$$k_c^2 = \eta(Db + 1)/2D. \tag{C}$$

In the three-dimensional case the nonlinear bifurcation analysis serves to describe the changes in the dynamics of the system around the linear stabilities when the parameters of the system vary. In the case of Turing systems the bifurcation analysis answers the question concerning the changes in the stability of different morphologies. We thus take a simple cubic lattice for exploring the possibilities for the structure of the patterns. The structures that arise from this analysis may get planar, cylindrical or spherical droplet arrangements for $k_c = 0.85$ only. This condition predicts that the planar structures could be stable for c <0.361, the spherical shapes are stable for 0.361 < c < 0.589 and the square packed cylinders are stable for all c <0.650 [9, 10].

We aim to understand pattern formation on a fixed sphere of radius R for the purposes of our model. Thus, we need to quantify the effect of domain size on patterning. The linear stability analysis for a spherical domain yields the range of wave numbers for which a pattern will emerge [1]:

$$L(\mathbf{A}, \eta, D) < k^2 < M(\mathbf{A}, \eta, D, R),$$

where

$$L(\mathbf{A}, \eta, D) = \frac{D\eta R^2}{2}\left[\left(\frac{1}{D}+b\right) - \left\{\left(\frac{1}{D}+b\right)^2 - \frac{4|\mathbf{A}|}{D}\right\}^{1/2}\right] = \eta R^2 \Theta_0,$$

and

$$M(\mathbf{A}, \eta, D) = \frac{D\eta R^2}{2}\left[\left(\frac{1}{D}+b\right) + \left\{\left(\frac{1}{D}+b\right)^2 - \frac{4|\mathbf{A}|}{D}\right\}^{1/2}\right] = \eta R^2 \Theta_1.$$

In these equations $|\mathbf{A}| = b - ah$ at the equilibrium point $(u_0, v_0) = (0, 0)$.

l	δ range	δ	Spherical harmonics
**6**	0.61-1.16	**1.06**	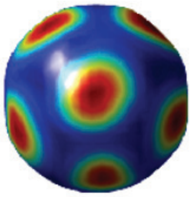
**5**	0.86-1.62	**1.58**	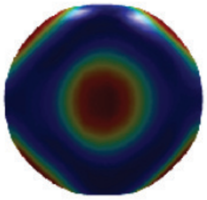
**4**	1.29-2.43	**2.30**	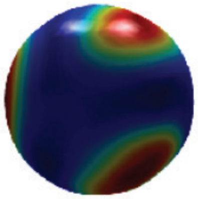
**3**	2.15-4.06	**3.45**	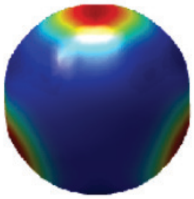
**2**	4.30-8.12	**4.46**	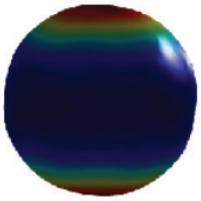

Figure A. Symmetries obtained on a sphere for different scales using the scale factor δ. The corresponding Turing pattern is related with the spherical harmonics l.

In the spherical symmetry, the associated Legendre equation shows that the eigenvalues l of the spherical harmonics are related to the wave modes as $k^2 = l(l+1)$ [6]. So, for every spherical harmonic and radius R we can find a Turing pattern if the domain size is changed by factor η/δ as:

$$\frac{\eta_0 R^2 \Theta_0}{l(l+1)} < \delta < \frac{\eta_0 R^2 \Theta_1}{l(l+1)}, \tag{D}$$

where $\eta_0 = 2Dk_c^2/(Db + 1)$.

To validate our numerical method, we performed simulations on a sphere of radius $R = 10$. We solved the equations of the chemical model (A) and (B) with the phase-field interaction (Equations 19 and 20 of the manuscript) for different values of δ. The amount of added mass was $m = 0$ every time and the other parameter values were the same as in the main text.

The symmetries on a sphere are highly correlated with the scale of the domain and also have an excellent matching with the reports of Turing pattens [5, 11]. The values of δ were chosen for different symmetries with the idea that the wave modes $k^2 = l(l + 1)$ were the nearest possible to the ideal value for a given symmetry. Thus for example, the range of δ to obtain the symmetry $l = 4$ is from 1.29 to 2.43, and we chose $\delta = 2.30$. In Fig A we present the values of δ and the corresponding Turing pattern.

References

1. Gjorgjieva J, Jacobsen J. Turing patterns on growing spheres: the exponential case. Discrete Continuous Dyn Syst Ser B. 2007;supp:436–445.
2. Lázaro G, Pagonabarraga I, Hernández-Machado A. Phase-field theories for mathematical modeling of biological membranes. Chem Phys Lipids. 2015;185:46–60.
3. Campelo F, Hernández-Machado A. Shape instabilities in vesicles: A phase-field model. Eur Phys J Spec Top. 2007;143(1):101–108.
4. Barrio RA, Varea C, Aragón JL, Maini PK. A two-dimensional numerical study of spatial pattern formation in interacting Turing systems. Bull Math Biol 1999;61(3):483–505.
5. Aragon JL, Torres M, Gil D, Barrio RA, Maini PK. Turing patterns with pentagonal symmetry. Phys Rev E. 2002;65:051913.
6. Gjorgjieva J. Turing pattern dynamics for spatiotemporal models with growth and curvature [Ph.D. Thesis]. Harvey Mudd College; 2006.
7. Campelo F. Shapes in Cells [Ph.D. Thesis]. Universitat de Barcelona; 2008.
8. Safran SA. Statistical thermodynamics of surfaces, interfaces, and membrane. No. 90 in Frontiers of Physics. Addison-Wesley; 1994.
9. Leppänen T, Karttunen M, Kaski K, Barrio RA. Dimensionality effects in Turing pattern formation. Int J Mod Phys B. 2003;17(29):5541–5553.
10. Kaski K, Barrio RA, editors. Current Topics in Physics in Honor of Sir Roger Elliot. Imperial College Press; 2005.
11. Varea C, Aragón JL, Barrio RA. Turing patterns on a sphere. Phys Rev E. 1999;60:4588–4592.
